# TRCCBP: Transformer Network for Radar-Based Contactless Continuous Blood Pressure Monitoring

**DOI:** 10.3390/s23249680

**Published:** 2023-12-07

**Authors:** Xikang Jiang, Jinhui Zhang, Wenyao Mu, Kun Wang, Lei Li, Lin Zhang

**Affiliations:** 1 School of Artificial Intelligence, Beijing University of Posts and Telecommunications, Beijing 100876, China; jiangxikang@bupt.edu.cn (X.J.);; 2 Logistic Support Center, Chinese PLA General Hospital, Beijing 100853, China

**Keywords:** contactless continuous monitoring, blood pressure, IR-UWB radar, pulse wave, transformer

## Abstract

Contactless continuous blood pressure (BP) monitoring is of great significance for daily healthcare. Radar-based continuous monitoring methods typically extract time-domain features manually such as pulse transit time (PTT) to calculate the BP. However, breathing and slight body movements usually distort the features extracted from pulse-wave signals, especially in long-term continuous monitoring, and manually extracted features may have limited performance for BP estimation. This article proposes a ***T***ransformer network for ***R***adar-based ***C***ontactless ***C***ontinuous ***B***lood ***P***ressure monitoring (TRCCBP). A heartbeat signal-guided single-beat pulse wave extraction method is designed to obtain pure pulse-wave signals. A transformer network-based blood pressure estimation network is proposed to estimate BP, which utilizes convolutional layers with different scales, a gated recurrent unit (GRU) to capture time-dependence in continuous radar signal and multi-head attention modules to capture deep temporal domain characteristics. A radar signal dataset captured in an indoor environment containing 31 persons and a real medical situation containing five persons is set up to evaluate the performance of TRCCBP. Compared with the state-of-the-art method, the average accuracy of diastolic blood pressure (DBP) and systolic blood pressure (SBP) is 4.49 mmHg and 4.73 mmHg, improved by 12.36 mmHg and 8.80 mmHg, respectively. The proposed TRCCBP source codes and radar signal dataset have been made open-source online for further research.

## 1. Introduction

Continuous blood pressure (BP) monitoring is of great significance for the prevention of hypertension in healthcare applications. Besides the commonly used sphygmomanometer that needs to apply relatively high pressure from the skin to local arteries, methods based on biosensors have gained attention in academia [1]. Wearable sensors such as photoplethysmography (PPG) have been researched to predict coronary artery diseases (CADs) [2] and heart diseases [3]. Amjed S.Al Fahoum [2] used PPG signals and featured selection-based classifiers to identify cardio-respiratory disorders based on the extraction of time-domain features. Existing studies utilizing PPG rely on pulse transit time (PTT) or pulse arrival time (PAT) for BP estimation [4,5]. However, these methods require attaching sensors to the human body surface, which can cause discomfort to participants. Recent advancements in remote photoplethysmography (rPPG) have opened up new possibilities for contactless continuous BP measurement. rPPG is a technique that utilizes the specular and diffused components of incident light, captured by optical devices, such as camera sensors, to measure physiological signals. Several remote techniques have been developed to estimate heart rate [6] or BP [7,8,9] from rPPG signals. However, these rPPG-based methods are highly sensitive to the lighting condition in environments and camera sensors may have privacy-concerning issues, thus limiting their usage in daily healthcare applications.

Compared to rPPG, a radar does not rely on lighting conditions and preserves privacy. Moreover, a radar with a wide bandwidth, such as the Impulse Radio Ultra-WideBand (IR-UWB) radar, is sensitive to micro cardiac and pulmonary motions such as respiration, heartbeat and arterial pulse. Pulse wave resulting from arterial pulse has been exploited to estimate blood pressure in recent years. Y. Ma [10] proved the relation between blood pressure and pulse wave velocity for human arteries. H.U. Chuan [11] monitored the skin vibrations and extracted pulse waves to estimate BP using electrode patches that attached to the skin. Since arterial pulse waves introduce vibrations on the body surface that could be carried on by a reflected radar signal, it is feasible to estimate BP using a radar. In recent years, several radar-based BP measurement methods have been proposed. Z. Zheng [12] estimated BP in a contactless manner with two continuous-wave (CW) radars. M. Kuwahara [13] proposed a BP estimation method based on a Doppler radar and a piezoelectric finger pulse sensor. M.-C Tang [14] detected wrist and chest movements using a CW radar. In [15], PTT was extracted from the small displacement on the body surface induced by the central aortic artery to estimate BP. T. Ohata [16] used a Doppler radar to obtain the cycle of cardiac dilation and contraction for BP estimation. With the advancement of deep learning technology, various methods automatically extract signal features through neural networks to calculate blood pressure [17,18,19]. S. Ishizaka [20] obtained clean pulse waves strongly correlated with PTT and BP using a deep learning model with long short-term memory (LSTM). However, due to breathing and slight body movements, pulse waves are distorted and have a low signal-to-noise ratio (SNR), especially in long-term continuous monitoring. Extracting features directly from distorted pulse waves is challenging. Therefore, contactless BP measurement methods often require participants to lie on a bed or even hold their breath, making continuous BP monitoring difficult.

**Figure 1:**
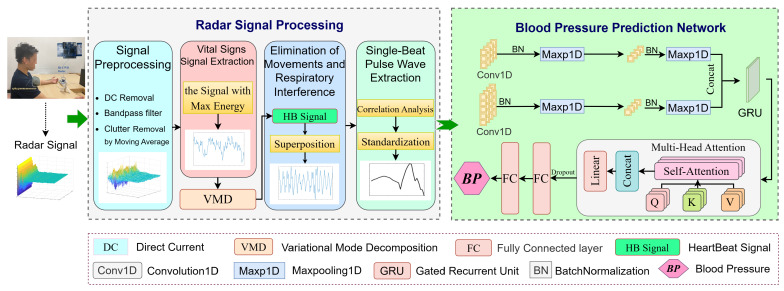
Flowchart of the proposed TRCCBP. The BP extraction method mainly consists of two parts: radar signal processing and blood pressure estimation network. After signal preprocessing and single-beat pulse wave extraction, the radar signal is denoised and the single-beat pulse wave is extracted as the input into the proposed blood pressure estimation network. Eventually, the output of TRCCBP is the estimated BP value extracted from the radar signal during a certain period of time.

In this article, a ***T***ransformer network for ***R***adar-based ***C***ontactless ***C***ontinuous ***B***lood ***P***ressure monitoring (TRCCBP) is proposed, as shown in Figure 1. After signal pre-processing, we select the column with the maximum energy in the radar matrix as the vital signs signal. Then, we remove the interference of breathing and slight body movements based on the consistency of heartbeats and pulses, and recover pulse-wave signals using variational mode decomposition (VMD). Subsequently, we choose single-beat pulse waves strongly correlated with the heartbeat signals as the input for the transformer network-based blood pressure estimation network. The network utilizes convolutional layers with different scales and a gated recurrent unit (GRU) to capture time-dependence in continuous radar signals. The deep temporal domain characteristics are captured through the multi-head attention module. Finally, extracted features are mapped to BP using fully connected (FC) layers. We have conducted experimental verification in two different scenarios: an indoor environment and a real medical environment. Results from both scenarios demonstrate that TRCCBP achieves high accuracy in blood pressure estimation and enables contactless continuous blood pressure monitoring. The contributions of this work are as follows:

1. We present a heartbeat signal-guided single-beat pulse wave extraction method that differs from existing denoising methods. It effectively extracts pure pulse-wave signals and provides a basis for blood pressure estimation.
2. We propose a transformer-based blood pressure estimation network suitable for processing continuous temporal radar signals. It can automatically capture appropriate time-domain characteristics of radar signals and map them to BP.
3. We have established an IR-UWB radar signal dataset for blood pressure measurement captured in an indoor environment and a real medical situation, which includes radar signal data and corresponding BP values from 36 persons in total. The proposed TRCCBP source codes and radar signal dataset are available at https://github.com/bupt-uwb/TRCCBP (accessed on 11 October 2023).

The remainder of this article is organized as follows. Section 2 describes the methods of TRCCBP. Section 3 presents the experimental setup and dataset. Section 4 presents experimental results and analysis. Section 5 concludes the article.

## 2. The Proposed TRCCBP Method

### 2.1. Signal Model

The IR-UWB radar periodically transmits narrow impulse signals with a wide bandwidth. The received signal can be expressed as the sum of the channel’s response and variation caused by vital signs:

(1) $$r\left(t,\tau \right)=\sum_{i}a_{i}p\left(\tau -\tau_{i}\right)+a_{v}p\left(\tau -\tau_{d}\left(t\right)\right),$$

where *t* is the pulse accumulative time, τ is the pulse sampling time, p(τ) is the transmitted pulse, $a_{i}$ is the amplitude of each multipath component and $a_{v}$ is the amplitude of the vital signs. $\tau_{i}$ and $\tau_{d}$ denote the time delay in the process of signal transmission and reception, and $\tau_{d}\left(t\right)=\frac{2d_{c}\left(t\right)}{c}$.

The received radar data are stored in the form of matrix $R\left[n,m\right]$ after sampling:

(2) $${R\left[n,m\right]}^{\prime }=r\left(nT_{s},mT_{f}\right)-\frac{1}{M}\sum_{i}^{M}r\left(nT_{s},iT_{f}\right),$$

where $T_{s}$ and $T_{f}$ are the sampling intervals in slow-time and fast-time, respectively. Each row of matrix *R* represents the *n*-th received frame with M fast-time sampling points (n=1,2,3,…,N;m=1,2,3,…,M). The signal propagation environment is static, and the movements in the environment are caused by human activities. To distinguish the static components of the radar signal from the dynamic components, the first step is to remove the average value of the signal, also known as the DC offset, from the received signal. This removes ambient static echoes that may interfere with the dynamic components of the signal.

After subtracting the DC offset, the signal is then filtered with a band-pass filter that matches the radar’s operating band, which is 6–8.5 GHz of the Novelda X4M03 IR-UWB radar (NOVELDA Oslo in Oslo, Norway) in Section 3. The filter helps to remove additional noise from the signal. To extract the human body signal from a raw data signal that may contain background noise and stationary clutter, the running average algorithm is used. This algorithm helps to generate a clutter-suppressed signal by subtracting the estimated clutter from the received raw data signal. The estimated clutter signal can be expressed as

(3) $$C\left[n,m\right]=\alpha C\left[n-1,m\right]+\left(1-\alpha \right){R\left[n,m\right]}^{\prime },$$

where $C\left[n,m\right]$ denotes the estimated clutter signal at the *n*-th slow-time, and α is the gain factor which can determine the renewing ratio of the clutter signal.

### 2.2. Heartbeat Signal-Guided Single-Beat Pulse Wave Extraction

In a static situation, the chest vibration resulting from heartbeat and breathing represents the primary source of energy. Consequently, the radar matrix identifies the column with the highest energy as the vital signs signal. This signal comprises the pulse wave, respiratory, and heartbeat signals, along with their corresponding harmonic waves and slight movements interferences. VMD can decompose the mixed signal into several modal signals with different frequencies [21], allowing for the separation of the pulse wave from these modal signals. The objective is to solve the following constrained variational problem:

(4) $$\begin{array}{c} L\left(\left\{\mu_{k}\right\},\left\{\omega_{k}\right\},\lambda \right)=\alpha \sum_{k}{∥\partial_{t}\left[\left(\delta \left(t\right)+\frac{j}{\pi t}\right)\times \mu_{k}\left(t\right)\right]e^{-j\omega_{k}t}∥}_{2}^{2}\\ +{∥f\left(t\right)-\sum_{k}\mu_{k}\left(t\right)∥}_{2}^{2}+\langle \lambda \left(t\right),f\left(t\right)-\sum_{k}\mu_{k}\left(t\right)\rangle , \end{array}$$

Here, $\mu_{k}$ represents the *k*-th modal signal, and $\omega_{k}$ denotes its center frequency. δ(t) is the Dirac function, $\partial_{t}$ is the partial differential to *t*, α is the quadratic penalty factor, λ is the Lagrange multiplier operator and f(t) is the input signal. The normal heart rate range is 0.8–2 Hz; thus, the modal signal with a center frequency within this range is considered as the heartbeat signal.

**Figure 2:**
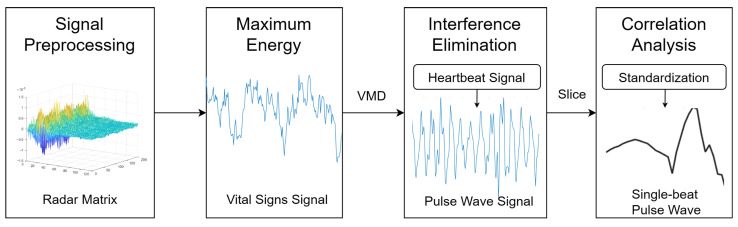
Heartbeat signal-guided single-beat pulse-wave extraction.

The aortic pulse wave has the same frequency as the heartbeat because it results directly from the heartbeat. However, its intensity is relatively low compared with the heartbeat signal, breath signal and other interferences. In order to obtain more characteristics related to blood pressure, the modal signals corresponding to breath, slight body movements and environmental noise derived from Equation (4) are removed from the vital signs signal. The remaining modal signals are added and considered as the pulse-wave signal. Considering that body movements and breathing correspond to modal signals with large energy, and stationary environmental noise corresponds to signals with the minimum energy, we arrange the decomposed signals from high to low energy in order first. Supposing that the heartbeat signal is the *n*-th signal, the pulse-wave signal can be expressed as

(5) $$f_{p}\left(t\right)=\sum_{i=n}^{m-1}\mu_{i}\left(t\right),$$

where $\mu_{i}\left(t\right)$ is the sorted *i*-th modal signal, and *m* is the number of modal signals decomposed from the original radar signal by the VMD algorithm. The first to (n−1)-th modal signals are signals with large energy which could be considered as breaths or slight body movements. The *m*-th signal is considered as environmental noise with lowest energy. The example of final extracted pulse-wave signal $f_{p}\left(t\right)$ is shown in Figure 2, which shows clear periodic characteristics.

In order to ensure the effectiveness of the extracted pulse-wave signal, we first slice the pulse wave and heartbeat signal with a certain length into several one-period signals. Specifically, we slice the pulse-wave signal based on the minimum points to obtain one complete single-beat heartbeat signal. However, single-beat pulse wave from signals of low SNR could be distorted severely and could not be utilized to estimate BP. Since the single-beat heartbeat signal is highly relevant to the single-beat pulse-wave signal, we could select the pulse waves with a high fitting degree as the input of the neural network by calculating the correlation coefficient between single-beat pulse waves and single-beat heartbeat signals. The correlation coefficient is calculated as

(6) $$r\left(g_{p},g_{h}\right)=\frac{\mathrm{Cov}(g_{p},g_{h})}{\sqrt{\mathrm{Var}\left[g_{p}\right]\mathrm{Var}\left[g_{h}\right]}},$$

where $g_{p}$ denotes the single-beat pulse-wave signal, $g_{h}$ denotes the single-beat heartbeat signal, Cov(·) is covariance formula and Var[·] is variance formula. Only single-beat pulse-wave signals with coefficients higher than a certain threshold could be reserved and others were abandoned due to distortion. In this article, the threshold is empirically set as 0.6.

### 2.3. Transformer Network-Based Blood Pressure Estimation Network

**Figure 3:**
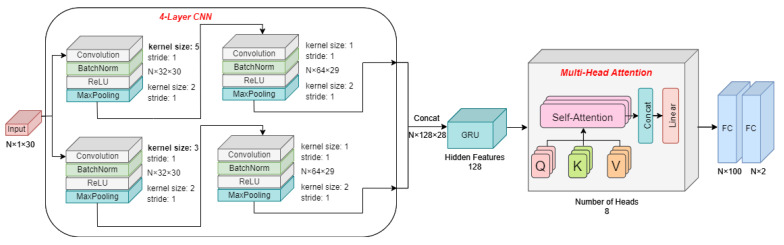
The architecture of the transformer network-based blood pressure estimation network.

The blood pressure estimation network architecture is shown in Figure 3. The network is trained in batch with a scale of N. The input of the network is 1×30 single-beat pulse-wave vectors for N batches. A four-layer CNN is introduced to capture the hidden features in the single-beat pulse wave. The input is fed into two convolution layers with different kernel sizes, which are 3 and 5, specifically. The small convolution kernel has a limited receptive field and can be utilized to dig into local features, while a larger convolution kernel has the ability to sense overall features. Each convolution layer is succeeded by a convolution layer with a kernel of size 1 to improve the network depth. The BatchNormalization (BN) layer is added before the activation layer ReLU to accelerate the convergence rate and prevent gradient explosion. Max-pooling layers with a kernel of size 2 are used to prevent over-fitting. The output features at two different scales are concatenated and fed into a one-layer gate recurrent unit with 128 features to improve the temporal expression ability.

Considering the insufficient effective information of the waves, the attention mechanism from the transformer network is adopted to assign appropriate weights to the temporal signal. Compared with traditional manual feature extraction methods, the attention mechanism effectively focuses on valuable features, enabling neural networks to focus on their feature subsets. Multi-head attention is from the encoder module in the transformer. It can capture the time-related information of features and is widely used in machine translation, natural language processing and other fields. The problem of the model excessively focusing its attention on its own position when encoding information about the current position will be solved. The module is formed by the combination of several self-attention layers. The significant parameters of the self-attention layer are matrices Query (Q), Key (K) and Value (V). The three matrices are obtained by linear transformation through the same input. Multi-head makes it possible to obtain information from different representation sub-spaces at different positions. Each head is similar to the self-attention layer. Then, the multi-head attention concatenates all the heads and obtains the output through a linear transformation. It can be expressed as

(7) $$\begin{array}{c} head_{i}=\mathrm{Score}\left(Q_{i},K_{i}\right)V_{i}=\mathrm{softmax}\left(\frac{QK^{T}}{\sqrt{d_{k}}}\right)V_{i}\\ \mathrm{Multi-head}\left(Q,K,V\right)=\mathrm{Concat}\left(head_{1},\ldots ,head_{n}\right)W^{o} \end{array}$$

where $d_{k}$ is the column number of Q. The final output is obtained by multiplying the score matrix and V. $W^{o}$ is a weight matrix which is used for linear transformation.

Finally, through two FC layers, the BP value for the corresponding period of time is estimated in the form of a vector with two elements, which are SBP and DBP.

## 3. Experimental Setting and Dataset

In the experiments, a commercial IR-UWB radar (X4M03, Novelda Inc., Oslo, Norway) is used to collect data in the real-world scenario. The transmitting antenna transmits short Gaussian pulses with a 7.29 GHz center frequency and a 1.4 GHz bandwidth. The sampling frequency is 23.328 GHz, which determines the sampling interval as 0.0064 m. Considering the amount of training samples and the actual situation, the number of received frames per second is set as 20 and the detection range is set as 0.2–3 m. For references, aN FDA-approved sphygmomanometer is utilized to collect blood pressure as the ground-truth value.

**Figure 4:**
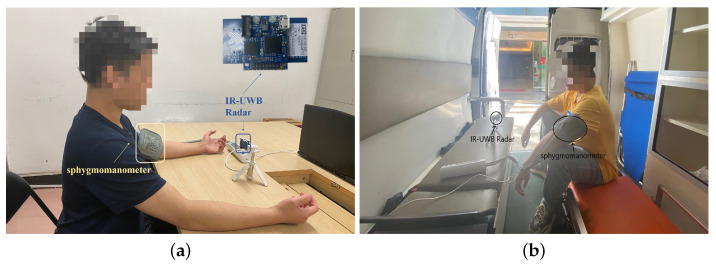
The experimental environment. (**a**) The indoor environment. (**b**) The stationary ambulance medical environment.

The experiments are conducted in two environments. The first environment is an indoor environment, as shown in Figure 4a. The indoor environment dataset was collected from 31 participants marked as ID 1 to 31. The physical conditions of these participants are summarized in Table 1. At one point in the data collection, they were required to sit still with their chests facing the radar placed 60 cm away. The sphygmomanometer was installed on the participant’s left arm and collected blood pressure values simultaneously. Radar signals of 30 s together with the corresponding blood pressure value were recorded during the time of collection. In order to demonstrate the applicability of TRCCBP in different physical situations, three scenarios were designed:

A. Relaxation: The participant was asked to breathe regularly during 30 s for data collection.
B. Apnea: During 30 s for data collection, the participant was asked to hold their breath for 10 s, then breathe regularly for 10 s and hold their breath again for 10 s.
C. Post-exercise: Before data collection, the participant was asked to run for 30 s to produce a hypertension state and then breathe rapidly during 30 s for data collection.

For participants whose IDs were from 1 to 11, they were asked to collect data in scenario A 30 times. For participants whose IDs were from 12 to 31, they were asked to collect data in scenario A 10 times, scenario B for 10 times and scenario C for 10 times. Based on the description in Section 2.2, we select a total of 27,679 effective single-beat pulse waves with a correlation coefficient higher than 0.6, accounting for 80.3% of the total. Considering that one single-beat pulse-wave cycle is typically no more than 1.5 s, which corresponds to 30 slow-time sampling points, we fill zero values at the end of each pulse-wave signal sequence to scrape 30 slow-time sampling points, and standardize pulse waves with the absolute maximum value of all valid pulse-wave data in three dataset as the input of the neural network. In the indoor dataset consisting of 31 individuals, we divide the data of 28 individuals (ID 1 to 28) as **Dataset 1** into training and testing sets with a ratio of 80%:20%. Additionally, we reserve the data from the remaining three individuals (ID 29, 30, 31) as **Dataset 2**, whose data are not included in the training set. In order to validate the blood pressure estimation ability of the TRCCBP algorithm for people in the real medical environment, we also collect the data of five new participants in a stationary ambulance (**Dataset 3**), as shown in Figure 4b. Each one was asked to collect data in scenario A 30 times. The physical conditions of five participants in this environment cannot be listed and their dataset cannot be open-source concerning privacy issues, while the radar signal data can be used for research purposes in our article after gaining permission. The data of Dataset 3 are also not included into the training set to demonstrate the generalization performance of the propose TRCCBP when facing a different environment.

Overall, a total of three datasets containing 36 persons in two environments were collected to evaluate the performance of the proposed TRCCBP method.

**Figure 5:**
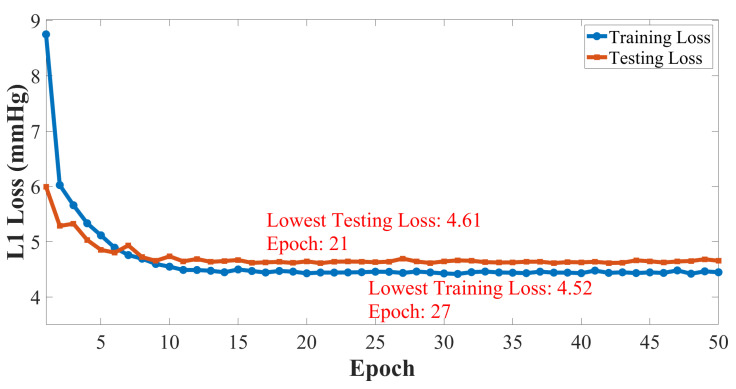
The architecture of transformer network-based blood pressure estimation network.

The processing terminal is a desktop server equipped with Intel Core i9-10940X CPU (main frequency 3.3 GHz, 28 cores), NVIDIA TITAN RTX graphics card (video memory 24 GB, 384 bit width, 32.62 TFLOPS for FP16 calculation) and 128 GB of running memory. The Adam optimizer is selected for the network training with a initial learning rate of 1 × 10${}^{4}$. We select the mean absolute error (MAE) as one of the evaluation indicators and the accuracy of SBP and DBP are equally important in BP estimation. Therefore, L1 Loss is selected as the loss function to calculate the average MAE of SBP and DBP between estimated BP values and their corresponding label. The batch size is set to 32 and the number of training epochs is 50. Dataset 1 is divided into training set and testing set with a ratio of 80%:20%. Variations in train loss and test loss on Dataset 1 over epochs are shown in Figure 5. The training and testing losses remain overall steady from the 10th training epoch. The lowest testing loss is 4.61 mmHg appearing in 21st epoch and the lowest training loss is 4.52 mmHg appearing in 27th epoch, which only differ by 0.09 mmHg. This indicates that the proposed TRCCBP network and the training setting could handle the over-fitting issues well.

The results of the ablation experiments are shown in Table 2, which show the importance of CNN, GRU and multi-head attention for the blood pressure estimation network. It can be concluded that each component of the TRCCBP algorithm has positive influences on the overall performance.

## 4. Experimental Results and Analysis

In this article, MAE and standard deviation error (STD) are used as the evaluation indicators. We select the average value of blood pressure estimated by the single-beat pulse waves of each participant as the estimated blood pressure. The evaluation results of the TRCCBP algorithm and method proposed by T.Ohata [16] based on three datasets are shown in Table 3. In Dataset 1, the average MAE with the TRCCBP algorithm of DBP is 4.49 mmHg, and that of SBP is 4.73 mmHg, which are 8.80 mmHg and 12.36 mmHg better than T.Ohata’s method. The average STD with the TRCCBP algorithm of DBP is 5.25 mmHg, and that of SBP is 5.52 mmHg. In Dataset 2, the results are 5.88 mmHg, 5.97 mmHg, 6.12 mmHg and 6.31 mmHg, respectively, which are slightly worse than that of Dataset 1 because the data in Dataset 2 are not included in the training set and the identities of three participants are different from the remaining twenty-eight participants in Dataset 1.However, in Dataset 3, because the multipath interference caused by the metal wall in the ambulance is relatively serious, the performance decreases, while the average MAE value still maintains a relatively low level compared with T.Ohata’s corresponding results. The results indicate that the TRCCBP algorithm can effectively predict BP for all of the participants in two different environments without prominent losses in estimation accuracy.

**Figure 6:**
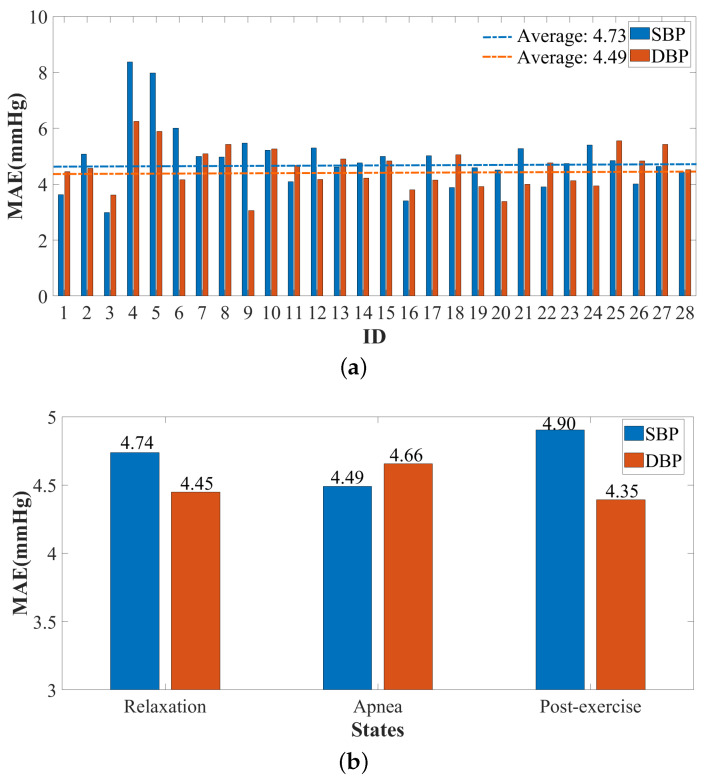
TRCCBP performance on Dataset 1 for different individuals and different states. (**a**) The MAE of TRCCBP blood pressure estimation for 28 participants. (**b**) The MAE of TRCCBP blood pressure estimation for 3 states.

Figure 6a,b, respectively, represent the performance of blood pressure estimation for each participant in Dataset 1 in the testing set and under different scenarios. The third participant has the lowest MAE for SBP (2.99 mmHg), and the ninth participant has the lowest MAE for DBP (3.05 mmHg). The fourth participant has the highest MAE for SBP (8.37 mmHg), and this participant also has the highest MAE for DBP (6.25 mmHg), so it is almost the same level as the fifth participant. This is mainly because the fourth and fifth participants are two females with relatively weak body conditions compared to the other participants. The SNRs of the reflected radar signals from them are much lower than those of others. This indicates that a certain kind of vital sign signal amplification algorithm should be considered in the future work. Moreover, it maintains a low MAE (<5 mmHg) in the test set in all three scenarios. It can be seen that TRCCBP exhibits good robustness in various states, including respiratory abnormalities (apnea) and hypertension (post-exercise), which meets the standard requirements of the FDA’s AAMI for a medical BP device.

**Figure 7:**
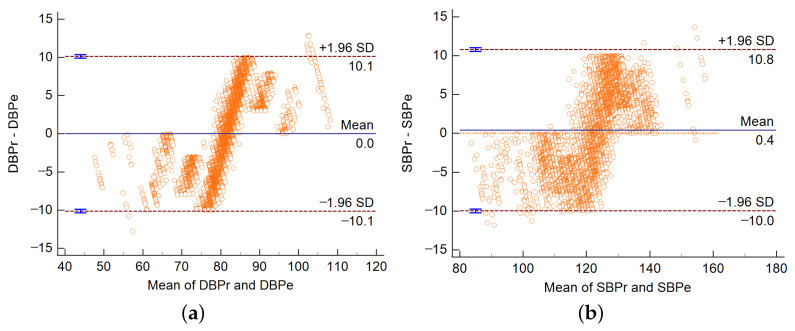
Bland-Altman plot of estimated blood pressure by TRCCBP. (**a**) DBP Bland–Altman plot. (**b**) SBP Bland-Altman plot.

For all the data in the test set, we use the Bland–Altman plot to judge the consistency of TRCCBP and the sphygmomanometer measurement method. As shown in Figure 7, DBPr and SBPr are ground-truth BP measured by a sphygmomanometer. DBPe and SBPe are blood pressure estimated by TRCCBP. The abscissa represents the mean of blood pressure from two methods. The ordinate represents the difference in blood pressure between two methods. SD is the standard deviation. The area covered by the dotted line is 95% limits of agreement. When most of the differences fall within the region and the mean value is close to zero, it shows that the two methods have good consistency. According to Figure 7, the TRCCBP algorithm and the measurement method by a sphygmomanometer have good consistency.

**Figure 8:**
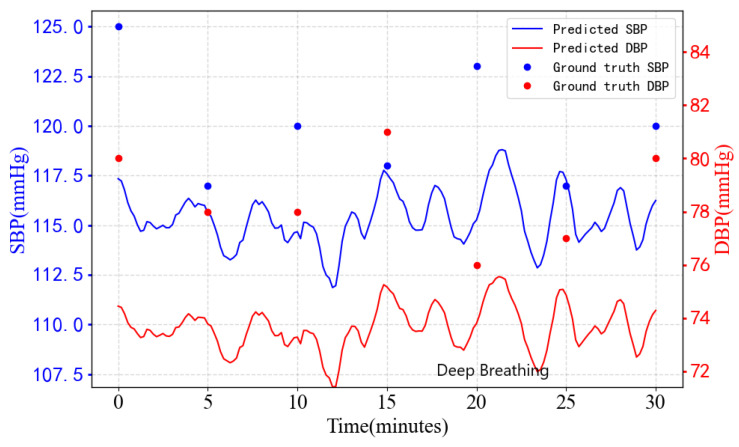
Blood pressure variations for 1 participant for 30 min.

Figure 8 shows the results of the continuous monitoring of BP by TRCCBP for 30 min. From the figure, we can observe that the participants’ deep breathing activities resulted in changes in blood pressure (at the twentieth minute). Deep breathing causes a gradual decrease in blood pressure from over a few minutes up to 25 min, which is consistent with the changes depicted in the figure on predicted SBP and DBP, which all decrease over the following few minutes. This indicates that TRCCBP has the potential for long-term contactless continuous BP monitoring. However, the MAE of long-term BP monitoring is relatively high. This is mainly because big body movements such as twists and turns of the body lead to interference in the extracted signal of a single-beat heart, which should be considered and optimized in the future work.

## 5. Conclusions

Contactless continuous blood pressure monitoring holds great significance for disease prevention in indoor healthcare applications. To address this challenge, this article proposes a transformer network for radar-based contactless continuous blood pressure monitoring named TRCCBP. The proposed approach involves pre-processing, vital sign signal extraction, elimination of movement and respiratory interference and extraction of the single-beat pulse-wave signal as input for the blood pressure estimation network. The estimation network utilizes convolutional layers with different scales, a GRU module and a multi-head attention module to extract deep time-domain features, which are then mapped to blood pressure values through a fully connected layer. To evaluate the proposed method, we have created a radar dataset comprising data from 36 individuals, with the potential for future expansion into a large-scale dataset. The average MAE of the TRCCBP algorithm for diastolic blood pressure estimation is 4.49 mmHg, and 4.74 mmHg for systolic blood pressure. These results demonstrate the high accuracy and robustness of TRCCBP in blood pressure estimation. It can be concluded from the results that the proposed TRCCBP still faces difficulties when addressing persons of relatively low SNR radar signal and changing environments. The proposed TRCCBP presents a promising approach for contactless continuous blood pressure monitoring, and further advancements can be achieved by expanding the dataset and exploring specific application scenarios.

## Figures and Tables

**Table 1:** Physical conditions of participants.

Demographics	Gender		Age		Height (cm)		Weight (kg)	
	Male	Female	>24	≤24	>175	≤175	>70	≤**70**
**Num**	20	11	10	21	16	15	18	13

**Table 2:** TRCCBP ablation experiments. A: TRCCBP, B: Using LSTM to replace GRU, C: Without Multi-head attention, D: Without GRU, E: Without CNN.

Methods (MAE)	A	B	C	D	E
SBP (mmHg)	4.73	5.19	6.49	6.96	8.05
DBP (mmHg)	4.49	4.82	5.21	5.54	6.43

**Table 3:** Blood pressure estimation performance comparison on three datasets.

		Dataset 1		Dataset 2		Dataset 3	
**Method**		**TRCCBP**	**T.Ohata**	**TRCCBP**	**T.Ohata**	**TRCCBP**	**T.Ohata**
SBP(mmHg)	MAE	4.73	13.53	5.97	14.14	7.75	17.20
	STD	5.52	12.64	6.31	13.82	6.40	15.20
DBP(mmHg)	MAE	4.49	16.85	5.88	15.97	6.85	11.88
	STD	5.25	16.55	6.12	16.35	6.35	10.98

## Data Availability

The source code and radar signal dataset are available at https://github.com/bupt-uwb/TRCCBP, accessed on 11 October 2023.

## Abbreviations

The following abbreviations are used in this manuscript:

PTT	Pulse Transit Time
VMD	Variational Mode Decomposition
FC	Fully Connected
IR-UWB	Impulse Radio Ultra-Wideband
CNN	Convolutional Neural Network
BN	BatchNormalization
SNR	Signal-to-Noise Ratio
MSE	Mean Square Error
LSTM	Long Short-Term Memory
GRU	Gated Recurrent Unit
BP	Blood Pressure
DBP	Diastolic Blood Pressure
SBP	Systolic Blood Pressure

## References

[1] Man P.K., Cheung K.L., Sangsiri N., Shek W.J., Wong K.L., Chin J.W., Chan T.T., So R.H.Y. (2022). Blood Pressure Measurement: From Cuff-Based to Contactless Monitoring. Healthcare.

[2] Al Fahoum A.S., Abu Al-Haija A.O., Alshraideh H.A. (2023). Identification of Coronary Artery Diseases Using Photoplethysmography Signals and Practical Feature Selection Process. Bioengineering.

[3] Neha, Sardana H., Kanwade R., Tewary S. (2021). Arrhythmia detection and classification using ECG and PPG techniques: A review. Phys. Eng. Sci. Med..

[4] Ganti V.G., Carek A.M., Nevius B.N., Heller J.A., Etemadi M., Inan O.T. (2021). Wearable Cuff-Less Blood Pressure Estimation at Home via Pulse Transit Time. IEEE J. Biomed. Health Inform..

[5] Herranz Olazabal J., Wieringa F., Hermeling E., Van Hoof C. (2023). Comparing Remote Speckle Plethysmography and Finger-Clip Photoplethysmography with Non-Invasive Finger Arterial Pressure Pulse Waves, Regarding Morphology and Arrival Time. Bioengineering.

[6] Mösch L., Barz I., Müller A., Pereira C.B., Moormann D., Czaplik M., Follmann A. (2023). For Heart Rate Assessments from Drone Footage in Disaster Scenarios. Bioengineering.

[7] Sugita N., Yoshizawa M., Abe M., Tanaka A., Homma N., Yambe T. (2019). Contactless technique for measuring blood-pressure variability from one region in video plethysmography. J. Med. Biol. Eng..

[8] Fan X., Ye Q., Yang X., Choudhury S.D. (2020). Robust blood pressure estimation using an RGB camera. J. Ambient Intell. Humaniz. Comput..

[9] Rong M., Li K. (2021). A blood pressure prediction method based on imaging photoplethysmography in combination with machine learning. Biomed. Signal Process. Control.

[10] Ma Y., Choi J., Hourlier-Fargette A., Xue Y., Chung H.U., Lee J.Y., Wang X., Xie Z., Kang D., Wang H. (2018). Relation between blood pressure and pulse wave velocity for human arteries. Proc. Natl. Acad. Sci. USA.

[11] Chung H.U., Rwei A.Y., Hourlier-Fargette A., Xu S., Lee K., Dunne E.C., Xie Z., Liu C., Carlini A., Kim D.H. (2020). Skin-interfaced biosensors for advanced wireless physiological monitoring in neonatal and pediatric intensive-care units. Nat. Med..

[12] Zheng Z., Wang B., Guo Y. Non-Contact Calibration-Free Blood Pressure Estimation Method Using Dual Radar. Proceedings of the 2022 IEEE MTT-S International Microwave Biomedical Conference (IMBioC).

[13] Kuwahara M., Yavari E., Boric-Lubecke O. Non-Invasive, Continuous, Pulse Pressure Monitoring Method. Proceedings of the 2019 41st Annual International Conference of the IEEE Engineering in Medicine and Biology Society (EMBC).

[14] Tang M.C., Liao C.M., Wang F.K., Horng T.S. Noncontact Pulse Transit Time Measurement Using a Single-Frequency Continuous-Wave Radar. Proceedings of the 2018 IEEE/MTT-S International Microwave Symposium-IMS.

[15] Zhao H., Gu X., Hong H., Li Y., Zhu X., Li C. Non-contact Beat-to-beat Blood Pressure Measurement Using Continuous Wave Doppler Radar. Proceedings of the 2018 IEEE/MTT-S International Microwave Symposium-IMS.

[16] Ohata T., Ishibashi K., Sun G. Non-Contact Blood Pressure Measurement Scheme Using Doppler Radar. Proceedings of the 2019 41st Annual International Conference of the IEEE Engineering in Medicine and Biology Society (EMBC).

[17] Schrumpf F., Frenzel P., Aust C., Osterhoff G., Fuchs M. (2021). Assessment of non-invasive blood pressure prediction from ppg and rppg signals using deep learning. Sensors.

[18] Jeong D.U., Lim K.M. (2021). Combined deep CNN–LSTM network-based multitasking learning architecture for noninvasive continuous blood pressure estimation using difference in ECG-PPG features. Sci. Rep..

[19] Wu B.F., Chiu L.W., Wu Y.C., Lai C.C., Chu P.H. Contactless Blood Pressure Measurement via Remote Photoplethysmography with Synthetic Data Generation Using Generative Adversarial Network. Proceedings of the IEEE/CVF Conference on Computer Vision and Pattern Recognition.

[20] Ishizaka S., Yamamoto K., Ohtsuki T. Non-contact Blood Pressure Measurement using Doppler Radar based on Waveform Analysis by LSTM. Proceedings of the ICC 2021-IEEE International Conference on Communications.

[21] Yang Z., Huang W. (2022). Wave Height Estimation From X-Band Radar Data Using Variational Mode Decomposition. IEEE Geosci. Remote Sens. Lett..

